# Functional Characterization of Duck STING in IFN-β Induction and Anti-H9N2 Avian Influenza Viruses Infections

**DOI:** 10.3389/fimmu.2019.02224

**Published:** 2019-09-18

**Authors:** Yuqiang Cheng, Yunxia Liu, Shuduan Shi, Qiaona Niu, Wenxian Zhu, Zhaofei Wang, Jingjiao Ma, Hengan Wang, Yaxian Yan, Jianhe Sun

**Affiliations:** Shanghai Key Laboratory of Veterinary Biotechnology, Key Laboratory of Urban Agriculture (South), Ministry of Agriculture, School of Agriculture and Biology, Shanghai Jiao Tong University, Shanghai, China

**Keywords:** duck, STING, IFN-β, antiviral immune response, AIV

## Abstract

The stimulator of interferon genes (STING) protein has been shown to play a pivotal role in response to both cytosolic RNA and dsDNA to elicit interferon (IFN) production in mammals. However, the role of duck STING (DuSTING) in antiviral innate immunity, especially in anti-RNA virus infection, has yet to be elucidated. In this study, the function of DuSTING in IFN induction and its role in anti-RNA virus infections were studied. DuSTING was amplified via reverse transcription-polymerase chain reaction (RT-PCR) from Pekin duck, showing that its cDNA sequence contains an open reading frame (ORF) of 1,149 bp and encodes 382 amino acids (aa). Sequence alignment showed that DuSTING protein shares 71.1, 43.4, and 33.3% identity with chickens, humans, and zebra fish, respectively. Overexpression of DuSTING in duck embryo fibroblasts (DEFs) strongly activated IFN-β promotor activity. Deletion mutant analysis revealed that the first 42 aa containing the first transmembrane (TM) domains and the last 32 aa containing a part of the C-terminal tail (CTT) are essential for its IFN-β activation. *In vitro* experiments showed that the mRNA levels of DuSTING and IFNs were all upregulated when the DEFs were infected with H9N2 avian influenza virus (AIV) SH010, while overexpression of DuSTING inhibited the replication of this virus. *In vivo* studies showed that DuSTING mRNA was widely expressed in different tissues, and was up-regulated in the spleen and lung of ducks challenged with SH010. In conclusion, our results indicate that DuSTING is an essential IFN mediator and plays a role in anti-RNA virus innate immunity.

## Introduction

Type I interferons (IFNs) play an essential role in innate immune responses against viral infection (1). The production of IFNs was elicited by a series of recognition molecules called cellular pattern recognition receptors (PRRs), such as the family of retinoic acid-inducible gene I (RIG-I)-like receptors (RLRs), the family of toll-like receptors (TLRs), and numerous DNA receptors (2, 3).

Although each PRR family includes many members, PRRs from the same family converge on the activation of one or two common junction adaptor proteins, which are essential for the subsequent signaling transduction (4, 5). For example, upon activation by a corresponding stimulus, TLRs converge on the activation of myeloid differentiation primary response gene 88 (MyD88) (6) or Toll/interleukin-1 receptor (TIR) domain-containing adaptor-inducing IFN-β (TRIF) (7), and RLRs converge on activating mitochondrial antiviral signaling protein (MAVS) (8, 9).

Recently, stimulator of the IFN gene (STING) was discovered as a multifaceted junction adaptor protein in the innate immune response that is involved in both DNA and RNA recognition signaling in mammals (10). For RNA recognition, STING utilizes RIG-I to recognize the RNA stimulus to trigger IFN signaling for inducing IFNs production; however, melanoma differentiation–associated gene 5 (MDA5), which belongs to the same family and shares a similar IFN pathway to RIG-I, cannot activate the IFNs via STING (10–12).

Chickens have a smaller repertoire of immune genes than mammals do (13). Many key immune genes, such as TLR8, TLR9, and IFN regulatory factor (IRF) 3, are missing in bird cells (14). Of especial note is that RIG-I, which plays a predominant role in RNA virus recognition and IFN induction, is also missing in chickens (15). However, although some essential immune genes are missing, chickens could initiate IFN response via a unique way. For example, although RIG-I is missing, Liniger et al. reported that chicken MDA5 could induce an IFNβ response via a classical MDA5-MAVS–IFN-β pathway to response to AIV infection (16). We also reported that chicken MDA5 could interact with STING to construct a MDA5-STING-IFN-β pathway (17), which is not presence in mammalian cells, for RNA viruses recognition. TLR8 is a pseudogene in chickens, is disrupted by several introns (18). However, the TLR7 gene which has a similar function as TLR8, which may partially takes over the function of TLR8, is presence in chicken cells (13). In addition, although TLR9 is not present in the chicken genome, TLR21, which is not observed in mammals is present and has a similar function (19).

Although some progress has got in the study about chicken IFNs signaling, the studies on duck IFN signaling are few. As a central and multifaceted junction adaptor protein, STING is involved in both DNA- and RNA-triggered IFN signaling, but its functions in innate immunity in ducks remain unclear. Chen et al. (20) reported that duck STING (DuSTING) can activate the IFN-β promoter with a luciferase reporter assay, and they demonstrated that DuSTING plays a role in DNA virus (duck plague virus) infection. More detailed works are needed to elucidate the functions of DuSTING in IFN induction. In addition, in the RLR intact duck cells, whether DuSTING could participate in anti-RNA viruses also remains unknown.

In this study, we provided more detailed experimental data to support the function of DuSTING in IFN-β activation. In addition, we investigated the function of DuSTING in RNA virus infection and illustrated that DuSTING plays a role in limiting H9N2 avian influenza virus (AIV) viral replication and infection. These results will help improve the general understanding of the biological role of STING in innate immunity and expand our knowledge on the relationship between STING and innate immunity in birds.

## Materials and Methods

### Cells and Virus

Duck embryo fibroblasts (DEFs) were prepared from 11-day-old duck embryonated eggs. DF-1, a chicken embryonic fibroblast cell line from East Lansing strain eggs, was cultured as in our previous study (21). The cells were maintained in complete Dulbecco's Modified Eagle Medium (DMEM; Hyclone, Logan, UT) supplemented with 10% fetal bovine serum (FBS; Life Technologies, Grand Island, NY) and incubated at 37°C in a 5% CO_2_ incubator. The A/Chicken/Shanghai/010/2008 (H9N2) virus (SH010) was isolated from chickens in Shanghai, China, in 2008. The viruses were purified, propagated, and stored as in our previous study (22).

### Cloning and Bioinformatics Analysis of DuSTING

Based on the predicted duck sequence from the National Center for Biotechnology Information (NCBI), the DuSTING-F and DuSTING-R primers (Table 1), which were located outside of the STING open reading frame (ORF), were designed and used to amplify potential DuSTING cDNA fragments by reverse transcription-polymerase chain reaction (RT-PCR) from total duck spleen RNA. The PCR product was cloned into a pTOPO-Blunt Cloning vector (Aidlab Biotech, Beijing, China), and the positive colonies were sent to the Beijing Genomics Institute (Beijing, China) for sequencing. The deduced amino acid sequences of DuSTING were analyzed using the SMART program. Amino acid sequences were aligned using Clustal X software and edited with BOXSHADE (https://embnet.vital-it.ch/software/BOX_form.html). Sequence homology and phylogenetic analysis of amino acid sequences used DNASTAR software. A phylogenetic tree was constructed from STING from 12 species.

**Table 1:** Primers used in this study.

**Primers**	**Purpose**	**Sequence (5^**′**^-3^**′**^)**
DuSTING-ORF-24 U	To obtain sequence	GGTGTCCTGGCCCTGGTCGCTCCG
DuSTING-ORF+30 L		GCGGGAGGCTCCTGCTGCAGGACG
*Eco*R I DuSTING U	Cloning	TAGTCCAGTGTGGTGUGAATTCATGTCTCAGGAACCGCAGCACCG
*Xho* I DuSTING L		GTCGTCCTTGTAGTCUCTCGAGGGGGTGGTCGCTCCGCAGGG
qDuSTING	qRT-PCR	ACAAGCACAGCCTCTACGCAATC
qDuSTING L		CGCAATGAGCCTGTAGGTTCC
qIFN-β U		CCTCAACCAGATCCAGCATT
qIFN-β L		GGATGAGGCTGTGAGAGGAG
qIL-1β U		TGGGCATCAAGGGCTACAAG
qIL-1β L		GCTGTCGATGTCCCTCATGAC
qIL-8 U		GAGCCTGGTAAGGATGGGAAA
qIL-8 L		CTGCGTCAGCTTCACATCTTG
qIRF1 U		AGCACCAACGACATCTACCAG
qIRF1 L		GAACTCCAACTCTGCCGAAG
qIRF7 U		GCCTGAAGAAGTGCAAGGTC
qIRF7 L		CTCTGTGCAAAACACCCTGA
DuSTING 1 U	Construct truncated forms of DuSTING	***CTCGAGGACTACAAG*** GACGACGATG
DuSTING 1-180 L		***CTTGTAGTCCTCGAG*** CTCCTTTATGCGTGGCAGAA
DuSTING 1-250 L		***CTTGTAGTCCTCGAG*** GATTGCGTAGAGGCTGTGCT
DuSTING 1-350 L		***CTTGTAGTCCTCGAG*** GCTCCCCTCGTACACCGTGA
DuSTING 1-368 L		***CTTGTAGTCCTCGAG*** GATCTGGAGGCTGAGATCTG
DuSTING 9-382 U		***AGTGTGGTGGAATTC** ATG* AGCAGCCCCGCTGCCCTGC
DuSTING 44-382 U		***AGTGTGGTGGAATTC** ATG* GAGCCCCTGTCCCCCGCTGC
DuSTING 85-382 U		***AGTGTGGTGGAATTC** ATG* GGCAGCTTCTGGAGGGCCCT
DuSTING 117-382 U		***AGTGTGGTGGAATTC** ATG* GGAGAGAGGCTCAGCCCCCA
DuSTING 149-382 U		***AGTGTGGTGGAATTC** ATG* GAGATGACCGAGAGGTCCCA
DuSTING 181-382 U		***AGTGTGGTGGAATTC** ATG* TGTATGGAGGAAATCAGCAG
DuSTING 382 L		***GAATTCCACCACACT*** GGACTAGTGG

*Homology arm sequences are highlighted in bold and italics. The restriction enzyme sites are underlined*.

### Plasmid Construction

The duck IFN-β promoter luciferase reporter plasmids pGL-IFN-β-Luc contain −390 to +63 of the duck IFN-β promotor motif (GenBank accession no. KM032183). Using PCR and the primers described in Table 1, the expression construct pcDNA-DuSTING-Flag was constructed by inserting full-length DuSTING into the pcDNA3.1-Flag vector via homologous recombination. The truncated forms of DuSTING, pcDNA-ST-1-180-Flag, pcDNA-ST-1-250-Flag, pcDNA-ST-1-350-Flag, pcDNA-ST-1-368-Flag, pcDNA-ST-9-382-Flag, pcDNA-ST-44-382-Flag, pcDNA-ST-85-382-Flag, pcDNA-ST-117-382-Flag, pcDNA-ST-149-382-Flag, and pcDNAST-181-382-Flag were constructed using a modified homologous recombination method with the primers in Table 1.

### qRT-PCR Analysis

Total RNA was extracted using the HP Total RNA Kit (OMEGA) and reverse transcribed into cDNA using a cDNA synthesis kit (Vazyme, Nanjing, China). A total of 1 μl of cDNA was amplified in 20-μl reactions using the Applied Biosystems 7500 Real-Time PCR system with the oligonucleotide primers listed in Table 1. The qRT-PCR mixture was composed of 10 μL of ChamQ SYBR qPCR Master Mix (Vazyme), 8.6 μL of nuclease-free water, 1 μL of cDNA, and 0.2 μL of each gene-specific primer (10 mM; Table 1). The relative expression levels of the tested mRNAs were determined using β-actin as an internal reference using the comparative Ct (2^−ΔΔ*Ct*^) method.

### Luciferase Reporter Assays

DEF or DF-1 cells were plated in 24-well plates and incubated until 90–95% confluence; they were then transiently transfected with reporter plasmid pGL-IFN-β-Luc (0.2 μg/well) and internal control *Renilla* luciferase (pRL-TK, 0.05 μg/well) along with the indicated plasmids using Hieff Trans (Yeasen, Shanghai, China). The cells were lysed 24 h post-transfection, and luciferase activity was measured using a dual reporter luciferase assay kit (Promega, Madison, WI) according to the manufacturer's instructions. *Renilla* luciferase activity was employed for normalization. All reporter assays were repeated at least three times.

### Virus Infection and qRT-PCR Analysis

For antiviral effect evaluation, DEF cells were transfected with pcDNA-DuSTING-Flag plasmid or empty plasmid. After 24 h, the transfected cells were washed twice and infected with 1 multiplicity of infection (MOI) of AIV. At 6, 12, and 24 h post-infection, supernatant aliquots were harvested for measuring viral titers as the 50% tissue culture infective dose (TCID_50_). The DEF cells were infected with AIV. After 24 h, qRT-PCR was performed for measuring the mRNA level of DuSTING and IFNs, as described above.

### Animal Experiments

Ducks used in this study were purchased from a duck farm and housed in isolators. Ducks were confirmed serologically negative for AIV by hemagglutinin inhibition assays. Twelve ducks were divided randomly into two groups of six. Group 1 was inoculated intranasally with SH010 AIV. Group 2 was inoculated intranasally with PBS as a control. At 1 and 2 days post infection (dpi), 3 ducks per group were killed and spleens and lungs were collected. The RNAs were extracted from the tissues and qRT-PCR were performed with the indicated primers (Table 1).

### Statistical Analysis

Student's *t*-test was used for determining the statistical significance of the differences. In addition, *p*-values of <0.05 were considered statistically significant.

## Ethics Statement

All the ducks used in this study have been conducted according to relevant national and international guidelines, and all efforts were made to minimize suffering. The study protocol was approved by Animal Ethics Committee of Shanghai Jiao Tong University.

## Results

### Duck STING Shares Low Similarity to Mammalian STING

**Figure 1:**
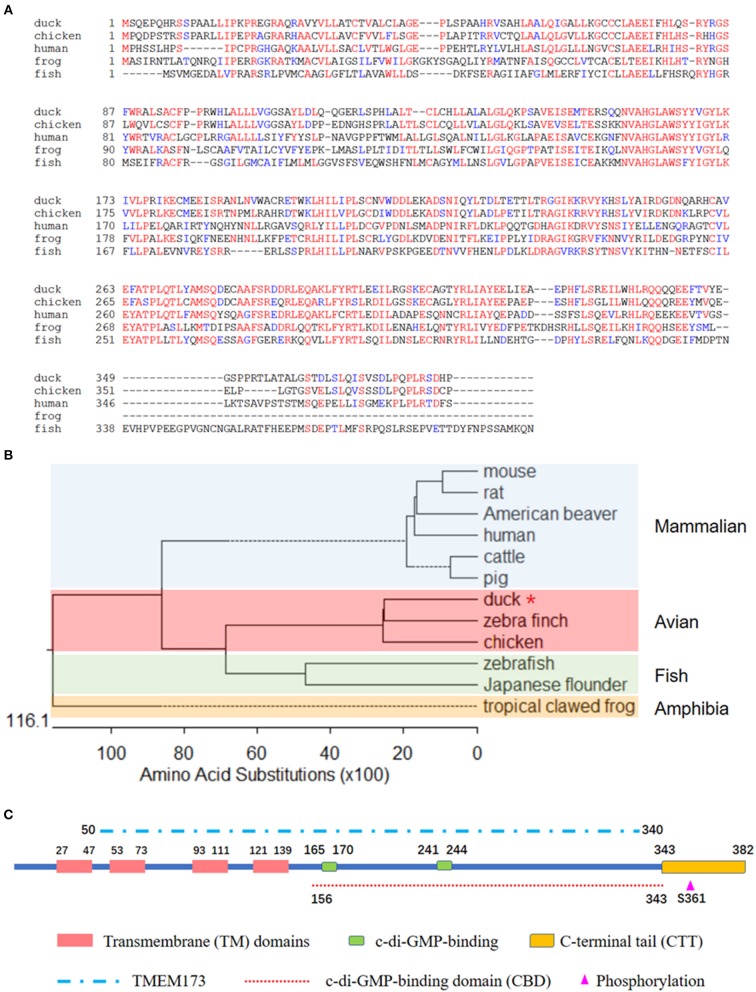
**(A)** Alignment of the deduced amino acid sequence of DuSTING with other animal STING proteins from the duck, chicken, human, frog, and fish. This was performed using the Clustal X program and edited with Boxshade. Red indicates amino acid identity and blue indicates similarity (50% threshold). **(B)** Phylogenetic tree of the deduced amino acid sequence of DuSTING and other animal STING proteins. The identified or predicted STINGs on the phylogenetic tree are sequences from different species available from the National Center for Biotechnology Information. The sequences were taken from GenBank entries, with accession numbers NP_082537.1 (mouse), NP_001102592.1 (rat), JAV42842.1 (American beaver), NP_938023.1 (human), NP_001039822.1 (cattle), AEL97644.1 (pig), XP_012430929.1 (zebra finch), NP_001292081.1 (chicken), BAU88509.1 (Japanese flounder), NP_001265766.1 (zebra fish), and NP_001106445.2 (tropical clawed frog). The number in the phylogenetic tree represents the bootstrap value. **(C)** The prediction of protein domains of duck STING.

To better understand the biological function of DuSTING, we cloned this gene from Pekin duck. The ORF of DuSTING contains 1,149 bp and encodes 382 amino acid (aa) residues (Figure 1A). Multiple sequence alignment showed that the amino acid sequences of DuSTING are 71.1, 43.4, and 33.2% identical to the STING gene in chickens (NP_001292081.1), humans (NP_938023.1), and zebra fish (NP_001265766.1), respectively. Phylogenetic analysis showed that the duck, chicken and zebra finch STING protein sequences were in the same subgroup. STING from fish, including Japanese flounder and zebra fish, were in another subgroup. The mammal STING sequences, including those of the mouse, rat, American beaver, human, cow, and pig were in the mammalian group. The tropical clawed frog was in an independent group, amphibia (Figure 1B).

Using the SMART program, along with the well establish protein domains of human STING, the protein domains of DuSTING were predicted. The four transmembrane (TM) domains, two c-di-GMP-binding sites and a C-terminal tail (CTT) domain, the TMEM173 (50–340 aa) domain, the c-di-GMP-binding domain (CBD, 156–343 aa), and the phosphorylation site are marked in Figure 1C.

### Overexpression of DuSTING Activates IFN-β Promoter

**Figure 2:**
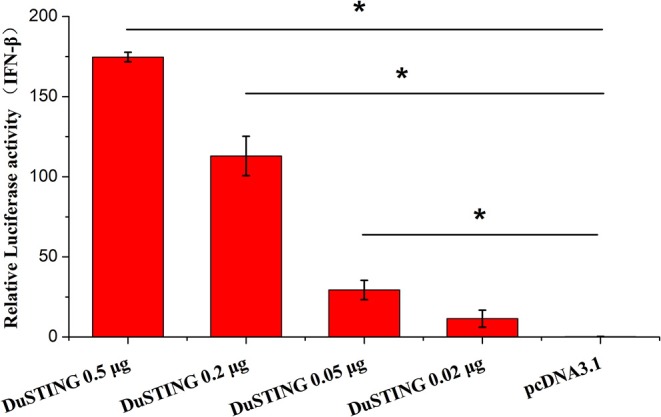
Overexpression of DuSTING activates the IFN-β promoter. DEF cells were transfected with an IFN-β promoter–luciferase reporter along with plasmid pcDNA-DuSTING-Flag (0.02, 0.05, 0.2, and 0.5 μg/well, respectively) or empty plasmid. Cells were lysed 24 h post-transfection, and luciferase activities were quantified by normalization with *Renilla* luciferase activity. The error bars are standard error of the mean (SEMs). Difference (*p < 0.05) between the experimental and control groups.

STING is a critical mediator of virus-triggered type I IFN signaling in RIG-I–null chicken cells. To investigate whether DuSTING is also involved in the type I IFN signaling pathway, we transfected DEF cells with constructs expressing DuSTING and the empty vector, respectively, and examined the IFN-β activation with a luciferase reporter assay. The results showed that the overexpression of DuSTING resulted in a remarkable activation of IFN-β promoter in DEFs, and the activation of IFN-β exhibited a positive correlation with the dosage of the DuSTING plasmid (Figure 2).

### Overexpression of DuSTING Induces Expression of IFN-β and ISGs

**Figure 3:**
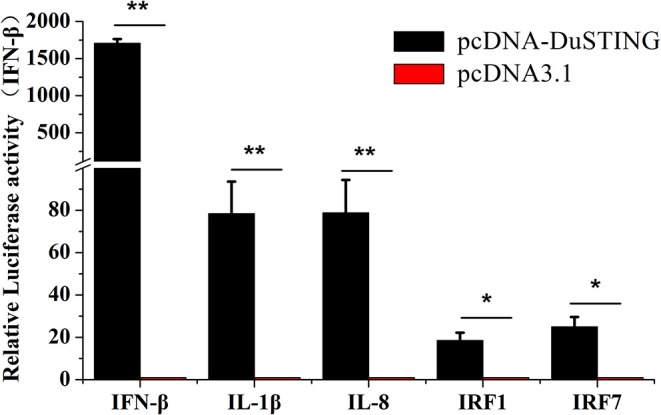
DEF cells respond to DuSTING overexpression. DEFs were transfected with pcDNA-DuSTING or empty plasmid (0.2 μg/well). At 24 h post-transfection, relative mRNA levels of IFN-β, proin?ammatory cytokines (IL-1β and IL-8), and interferon regulatory factors (IRF1 and IRF7) were analyzed by qRT-PCR. Results are for three independent experiments. Error bars are SEMs. Difference (*p < 0.05, **p < 0.01) between the experimental and control groups.

To understand immune induction by DuSTING, we examined mRNAs of IRF1 and IRF7, proinflammatory cytokines interleukin (IL)-1β, IL-8, and IFN-β via qRT-PCR. The expressions of all examined antiviral genes were significantly induced by overexpression of DuSTING (Figure 3). IFN-β mRNA showed the strongest expression, increasing by 1,707-fold (*p* < 0.05) compared with the empty vector–transfected control. IL-1β and IL-8 mRNA increased by 78.4-fold (*p* < 0.05) and 78.8-fold (*p* < 0.05), respectively, whereas IRF1 and IRF7 increased by 18.5-fold and 25.0-fold (*p* < 0.05), respectively. Thus, overexpression of DuSTING led to an induction of both IFN and IFN-stimulated genes (ISGs), as well as the expression of all examined antiviral molecule genes.

### The Essential Domains of DuSTING in IFN Activation

**Figure 4:**
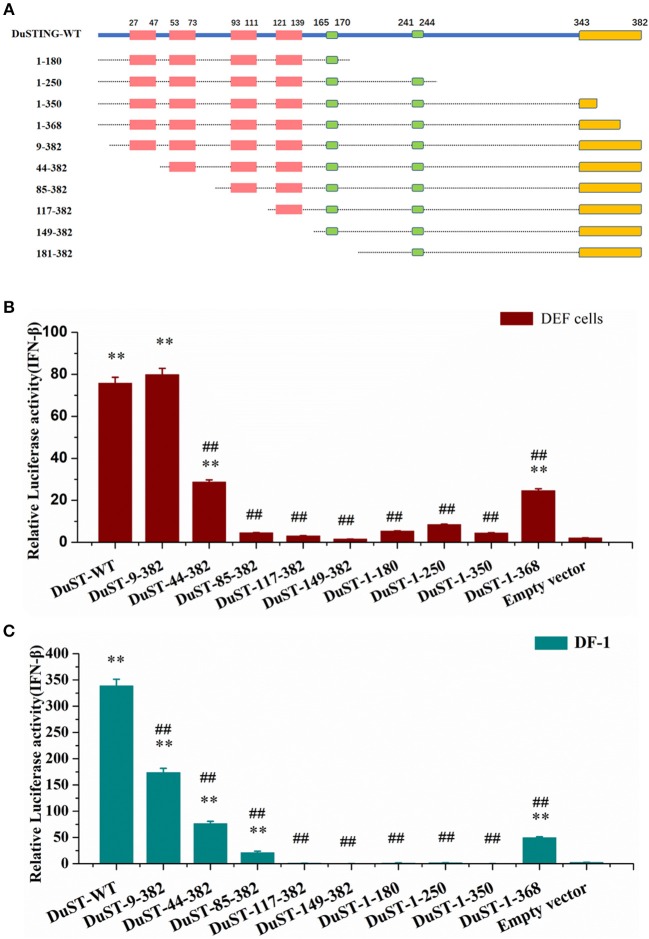
Essential domains of DuSTING for IFN-β promoter activity. **(A)** Schematic structure of DuSTING mutants. **(B,C)** IFN-β activity induced by DuSTING and its mutants in DEF or DF-1 cells. Cells were transfected with different expression plasmids of DuSTING (DuSTING-WT, DuSTING-1-180, DuSTING-1-250 DuSTING-1-350, DuSTING-1-368, DuSTING-9-382, DuSTING-44-382, DuSTING-85-382, DuSTING-117-382, DuSTING-149-382, or empty vector) together with the reporter plasmids pGL-IFN-β-Luc and internal control *Renilla* luciferase (pRL-TK). Luciferase assays were performed 24 h after transfection. (**p < 0.01 vs. empty vector; ##*p* < 0.01 vs. DuSTING-WT) All luciferase assays were repeated at least three times and the error bars are SEMs.

For identifying the essential domains of DuSTING in IFN-β activation, 14 DuSTING mutants lacking different function domains were constructed based on the DuSTING structure prediction diagram in Figures 1C, 4A. Their IFN-β activation abilities were assessed with the IFN-β luciferase report assay. The results showed that the deletion of the first 8 aa of the N-terminal of DuSTING did not affect its IFN induction in DEF. DuSTING-44-382, which lacks 43 aa at the N-terminal of DuSTING, showed a significant decrease compared with the wild-type DuSTING. DuSTING-85-382, DuSTING-117-382, and DuSTING-149-382, which lacks 84 aa more at the C-terminal, failed to activate the IFN-β promoter. For the N-terminal deletion truncations, DuSTING-1-368, even with a deletion of only 14 aa, led to a strong decrease in IFN-β induction. In contrast, DuSTING-1-180, DuSTING-1-250, and DuSTING-1-350, lacking 32 aa more, failed to activate the IFN-β promoter (Figure 4B).

Given that the poor transfection efficiency of the primary cells may affect the reliability and accuracy of the results, the same experiment was conducted with a chicken continuous cell line, DF-1 (Figure 4C), where most of its IFN pathways are conserved compared with that of ducks. A similar result was obtained to that in DEF, except in the DuSTING-9-382 mutants. Different from the DEF cells, deletion of the first 8 aa at the N-terminal significantly decreased the IFN-β activation in DF-1 cells.

### DuSTING Plays a Role in Anti-RNA Viruse Infection *in vitro*

**Figure 5:**
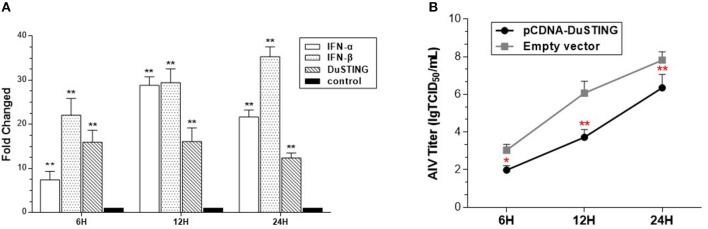
DEF cells respond to infection with SH010 AIV. **(A)** The DEF cells were infected with AIV. After 24 h, qRT-PCR was performed for measuring the mRNA level of DuSTING and IFNs (IFN-α and IFN-β). Fold expressions were calculated based on uninfected DEF cells. The results are for three independent experiments. **(B)** DEF cells were transfected with either the pcDNA-DuSTING-Flag or empty plasmid. After 24 h, cells were infected at 1 MOI by SH010. Supernatants were collected at the indicated timepoints and analyzed for TCID_50_ titers. Results are for three independent experiments. Error bars are SEMs. Difference (*p < 0.05, **p < 0.01) between the experimental and control groups.

To determine whether DuSTING could respond to the RNA virus and induce an IFN antiviral response, the expressions of DuSTING and IFNs were analyzed in DEF cells following infection with a H9N2 AIV SH010 using qRT-PCR. The results illustrated that the DuSTING, IFN-α, and IFN-β mRNAs in the AIV-infected DEF cells were all significantly elevated at 6, 12, and 24 h post-infection (Figure 5A). The data collected suggest that DuSTING may be involved in innate immune responses to AIV.

For evaluating the antiviral activity of DuSTING, the DuSTING-overexpressing or normal DEF cells were inoculated with SH010. The results showed that the viral titers of DuSTING-overexpressing DEF cells were lower than those of the control cells at all the tested timepoints, especially at 12 h (Figure 5B). This suggests that the overexpression of DuSTING in DEF cells allows for an innate immune response that reduces AIV replication.

### DuSTING mRNA Is Wildly Expressed in Different Tissues

**Figure 6:**
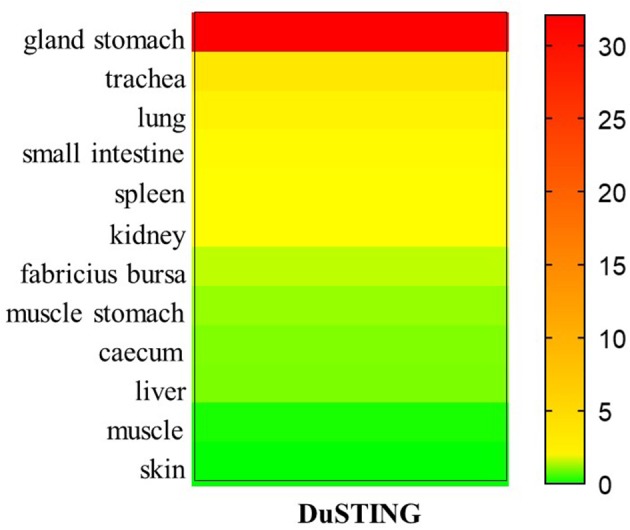
Relative expression levels of DuSTING mRNA transcripts in healthy duck tissues. Total RNAs extracted from different tissues of healthy duck and analyzed with real-time RT-PCR.

To better understand the tissue distribution of duSTING, The expression levels of duSTING mRNA in healthy duck tissues were analyzed by qRT-PCR. The DuSTING mRNA was widely expressed in all tissues analyzed (Figure 6). The largest quantity of DuSTING mRNA was found in glandular stomach, followed by the trachea, lung, small-intestine, spleen, kidney, bursa of fabricius, caecum, muscular stomach, and liver. The level of DuSTING mRNAs in muscle and skin was relatively low.

### DuSTING Is Upregulated by RNA Virus Infection *in vivo*

**Figure 7:**
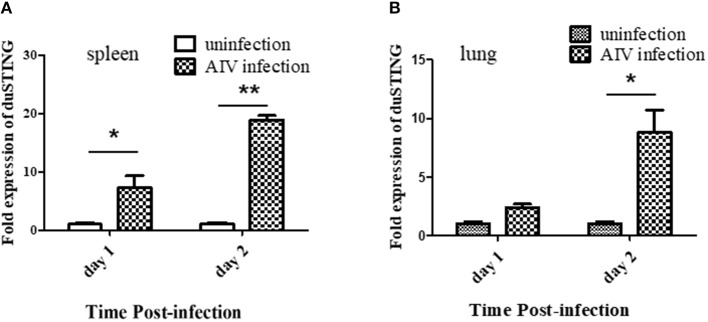
The expression profiles of DuSTING mRNA in virus-infected tissue by q RT-PCR. Relative DuSTING mRNA expression pattern in spleen **(A)**, and in lung **(B)**. The controls were inoculated with PBS; the experimental ducks were infected with H9N2 AIV. Each bar represents the level of target gene mRNA relative to those in control group. *The difference (*P < 0.05, **P < 0.01) between experimental group and control group. Error bars indicate SEMs.

Given that SH010 infection upregulated DuSTING mRNA *in vitro*, we further studied how DuSTING works *in vivo* after infection of SH010. DuSTING mRNAs in lung and spleen were detected following infection with SH010 by qRT-PCR. The result showed that the DuSTING mRNA was significantly up-regulated in spleen at both 1 and 2 day post-infection (Figure 7A). Though DuSTING mRNA level in lung was not increased significantly at 1 day-post-infection, it was significantly upregulated at 2 days post-infection (Figure 7B).

## Discussion

Although most of the signaling components and signaling pathways are conserved in chickens and ducks, differences are still evident between these two avian species. Especially, it has been discovered that ducks have an intact RIG-I gene, while chickens lack this gene (15), highlighting the importance of functional research on RLR in birds. In our previous study, we found that although RIG-I is missing in chickens, chicken MDA5 interacts with STING to construct a MDA5-STING-IFN-β pathway (17), which may be absent but replaced by an RIG-I-STING-IFN-β pathway in mammals (10, 11) for RNA sensing. Our aim was to determine the functions of DuSTING in innate immunity in ducks and find out whether DuSTING plays a role in RNA virus infection in RIG-I-present duck cells.

We firstly cloned DuSTING gene from Pekin duck. DuSTING cDNA contains 1,149 bp and encodes 382 aa residues (Figure 1A). The gene shares low nucleotide and amino acid sequence similarity with its counterparts. The amino acid similarities between DuSTING and mammalian STING range from 40.5 to 44.3% (Supplementary Figure 1). Even to its closest relative birds, zebra finches and chickens, the amino acid similarities were only 74.3 and 70.6%, respectively (Supplementary Figure 1).

STING was initially discovered as an IFN-activated gene where its overexpression could enhance IFNs production in mammals (11, 12, 23). So we then tested whether DuSTING was also involved in IFN activation. Result showed that overexpression of DuSTING strongly activated the IFN-β promoter in duck cells (Figure 2). In addition, with qRT-PCR assay, we also found that the mRNAs of IFN-β, proinflammatory cytokines (IL-1β and IL-8), and the interferon regulatory factors (IRF1 and IRF7) were all upregulated by DuSTING overexpression (Figure 3). The induction of the proinflammatory cytokines by DuSTING indicates that DuSTING had functions in addition to IFN mediator.

It is clear that STING activates IFNs depending on transcription factor IRF3 in mammalian cells (12). However, previous studies have shown that IRF3 is absent in ducks (13, 24, 25). How DuSTING activates IFNs in IRF3 missing ducks remains unclear. The upregulation expressions IRF7 by DuSTING overexpression enable us to speculate that DuSTING may activate IFNs via IRF7, which shows a high similarity in structure with IRF3 and belongs to the same subfamily with IRF3. More experimental evidences are needed to support this hypothesis.

STING has been reported to interact with RIG-I to constitute a RIG-I-STING-IRF3-IFN signaling, which may play a role in RNA recognition to enhance the IFN induction (17, 26). STING has also been reported to be negatively regulated by RIG-I in the process of DNA mediated IFN induction (27). Here, we cannot give a conclusion how RIG-I works for the function of DuSTING in RIG-I present ducks, based by the current data. Experiments based on a RIG-I knockout duck cell line may be needed to verify the function of RIG-I in STING signaling in ducks in the future.

After that we investigated the indispensable domain of DuSTING in IFN induction. A series of DuSTING mutants lacking a range of 8-148 residues at the N-terminal and l4-202 residues at C–terminal were generated; the mutants were transfected into both DEF and DF-1 cells, and the induction of IFN-β promoter activity was measured (Figure 4). The mutants were first transfected into DEF cells. The results showed that the deletion of 84 residues of DuSTING at the N-terminal, constituting the first two TM domains, abolished its IFN-β activation (Figure 4B). The TMs in mammalian STING were found to be essential for its mitochondria and endoplasmic reticulum localization (11, 23). The abolished IFN-β activation by DuSTING-85-382 may be due to the obstacle of its location to the organelle, which is a prerequisite for its IFN activation. The C-terminal deletion mutants of DuSTING made DuSTING defective in the CTT domain; they also failed to activate the IFN-β promoter, even with a deletion of only 38 aa (Figure 4B). This indicated that the CTT domain is another essential domain for IFN activation. The same experiment was conducted in the DF-1 chicken continuous cell line (Figure 4C). We found that the relative luciferase values in DF-1 were much higher than those in DEFs. Although the absolute values of the IFN activation in DF-1 and DEFs are different, a similar IFN activation tendency was obtained in these two cells, except the DuSTING-9-382 mutants.

Immune molecule is the basis of construction of signal transduction pathways. Although most of the immune molecules of chicken and duck show a high similarity, disparities still exist in IFN signaling between chicken and duck (28): (1) RIG-I is missing in chicken cells but presence in duck cells. (2) The amino acid sequence of IRF7, which locates downstream of STING and is strictly depended by STING in IFN induction production in birds, was found differ largely between chicken and duck (69.2%). The deletion of the first 8 amino from DuSTING may only affect its interaction with chicken IRF7 but not duck IRF7. We speculate that these and some other unknown differences in chicken and duck STING signaling may contribute to the different IFN induction by Du-STING9-382 in chicken and duck cells.

Increasing studies have shown that mammalian STING participates in both anti-DNA and RNA virus infections (10, 11, 29). Chen et al. reported that DuSTING plays a role in anti-DNA virus infection (20). Whether DuSTING could participate in anti-RNA viruses remains unknown. In the subsequent study, the function of DuSTING in anti-RNA viruses was to be studied using a H9N2 AIV as a model.

In SH010 AIV–infected DEFs, DuSTING, and the IFN mRNAs were upregulated (Figure 5A). Upregulation of gene expression is an important strategy for immune genes to defend against pathogens. The upregulation of DuSTING by AIV may indicate an essential role of DuSTING in response to this virus. In the subsequent research, we found that the titers of SH010 were much lower in DuSTING-overexpression DEFs than in the empty vector control group (Figure 5B). This suggests that the overexpression of DuSTING in DEF cells allows for an innate immune response that reduces AIV titers. All these findings indicated that DuSTING seems to play a role in combatting AIV infection. Previous reports showed that mammalian STING could respond to some RNA viruses, probably via the upstream adaptor RIG-I, but not MDA5 (11, 12, 23, 30). The chicken lacks the RIG-I gene (14, 15); even so, our previous study demonstrated that chicken STING can still respond to AIV through a complementary “MDA5-STING-IFN-β” signaling pathway. In this study, DuSTING was also found to be involved in anti-AIV infections. Ducks have an intact RIG-I, and duck MDA5 shows a high similarity with that of chickens. Which sensor DuSTING relies on to recognize AIV is unclear. Further studies are urgently needed to elucidate this point.

In this study, DuSTING was cloned from Pekin duck, and it was confirmed to be involved in the regulation of IFN-β induction. In addition, the essential domains of DuSTING required for the induction of IFN-β were determined. The expression of DuSTING was upregulated by a H9N2 AIV–infected DEFs, and its overexpression inhibited the replication of the H9N2 AIV. *In vivo* studies also showed that DuSTING could be upregulated response to AIV infections. Together, these results indicated that DuSTING is an important regulator of duck innate immune signaling, and it may be involved in anti-RNA virus infections. The results will further improve our understanding of the function of DuSTING in the regulation of IFN-β and its role in defending against RNA virus infections.

## Author Contributions

JS and YC designed the experiment. YC, YL, and SS performed the majority of the experiments. QN, WZ, ZW, and JM helped with the experiments. YC and YL wrote the paper. HW and YY helped analyze the experimental results.

### Conflict of Interest Statement

The authors declare that the research was conducted in the absence of any commercial or financial relationships that could be construed as a potential conflict of interest.

## Supplementary Material

Supplementary Figure 1 Sequence similarity analysis of STING among different species. The program was performed using MegAlign software. The sequences were taken from GenBank entries with the following accession numbers: mouse (NP_082537.1), rat (NP_001102592.1), American beaver (JAV42842.1), human (NP_938023.1), cattle (NP_001039822.1), pig (AEL97644.1), zebra finch (XP_012430929.1), chicken (NP_001292081.1), Japanese flounder (BAU88509.1), zebra fish (NP_001265766.1), and tropical clawed frog (NP_001106445.2).
