# Supplementary figures and images for: Functional Characterization of Duck STING in IFN-β Induction and Anti-H9N2 Avian Influenza Viruses Infections
Source: Front Immunol. 2019 Sep 18;10:2224. doi: 10.3389/fimmu.2019.02224 (PMC6759682; doi:10.3389/fimmu.2019.02224)

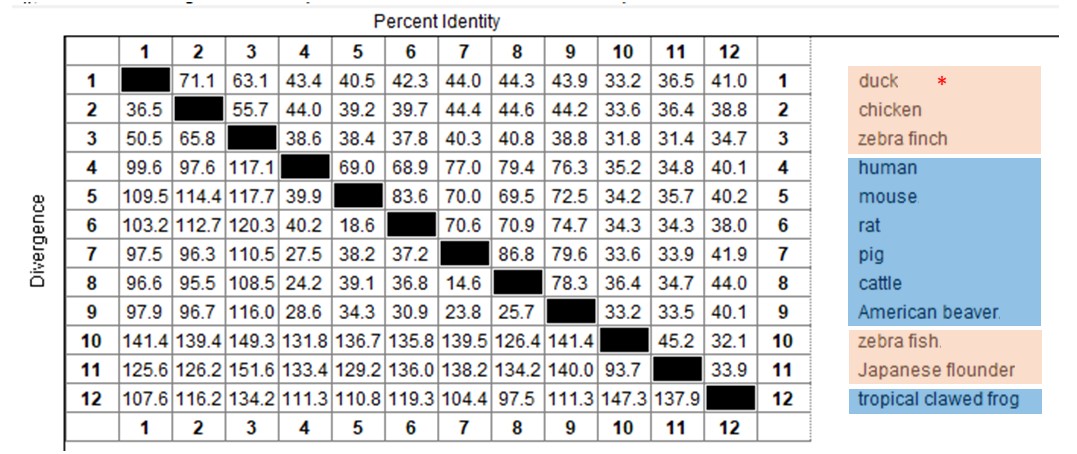

Supplement: Supplementary Figure 1 — Sequence similarity analysis of STING among different species. The program was performed using MegAlign software. The sequences were taken from GenBank entries with the following accession numbers: mouse (NP_082537.1), rat (NP_001102592.1), American beaver (JAV42842.1), human (NP_938023.1), cattle (NP_001039822.1), pig (AEL97644.1), zebra finch (XP_012430929.1), chicken (NP_001292081.1), Japanese flounder (BAU88509.1), zebra fish (NP_001265766.1), and tropical clawed frog (NP_001106445.2). [file Image_1.JPEG]
